# Mangrove mapping and monitoring using remote sensing techniques towards climate change resilience

**DOI:** 10.1038/s41598-024-57563-4

**Published:** 2024-03-23

**Authors:** Reshma Sunkur, Komali Kantamaneni, Chandradeo Bokhoree, Upaka Rathnayake, Michael Fernando

**Affiliations:** 1 University of Technology Mauritius, Port Louis, Mauritius; 2 University of Central Lancashire, Preston, England; 3 United Nations-SPIDER-UK Regional Support Office, University of Central Lancashire, Preston, UK; 4 Atlantic Technological University, Sligo, Ireland

**Keywords:** Climate change, Mangroves, Remote sensing, GIS, Mauritius, Natural hazards, Environmental impact

## Abstract

Mangroves are amongst the richest ecosystems in the world providing valuable goods and services to millions of people while enhancing the resilience of coastal communities against climate change induced hazards, especially island nations. However, these mangroves are severely affected by many anthropogenic activities. Therefore, understanding the spatial variability of mangroves in island nations is highly essential in the events of ongoing climatic change. Thus, this study assessed the use of remote sensing techniques and GIS to map and monitor mangrove cover change at selected sites, namely Le Morne and Ferney, on the tropical island of Mauritius. Freely available 2013 SPOT-5 and 2023 Sentinel 2A images were retrieved and processed using ArcGIS Pro tools and SNAP; mangroves were mapped based on Google Earth Pro historical imagery and ground truthing at the respective sites. Following the application of selected vegetation indices, GLCM and PCA analysis, mosaicked images were classified using the Random Trees algorithm. Kappa values of all the classified images were in the 90 s; Le Morne showed a significant increase in mangrove cover over the decadal scale with main class change from mudflat to mangroves. This study demonstrates how geo-spatial tools are crucial for monitoring mangroves as they provide spatially explicit and time sensitive information. Decision makers, researchers, and relevant stakeholders can utilize this data to bolster tailored mitigation and adaptation strategies at specific sites, thereby enhancing resilience to climate change.

## Introduction

Mangrove forests are amongst the richest ecosystems in the world^1^ growing mainly along coastlines, riverbanks and mudflats in the tropical and subtropical zones^2^. They provide vital goods to coastal populations such as food, honey, timber, wax while also serving as sources of income through fishing and tourism^3^; they also reduce the impacts of coastal hazards like storm surges, high swells and tsunamis^4^. Today, as climate change takes its toll on the global population, healthy mangrove ecosystems are viewed as ecosystems of hope to store carbon and build resilience against looming coastal hazards^5^. In fact, mangroves are now recognized as one of the most effective nature based solutions for climate change adaption and to reduce disaster risk^6^. Nevertheless, mangroves face severe terrestrial and oceanic pressures, natural and anthropogenic, because of their particular location in the intertidal zone^5^. Friess^7^ remark that since colonization of foreign land commenced in the nineteenth century, mangroves have been regarded as dark and gloomy, harbouring pests and vectors that carry diseases like malaria. These perceptions would influence views of mangroves down the years causing mass deforestation to make way for more profitable trade-offs such as resorts, settlements, agriculture, aquaculture, industry and mining^8–10^. However, following the devastating tsunami that hit the Indian Ocean in 2004 affecting 14 countries and killing over 200,000 people, studies showed that healthy and dense mangroves protected several areas^11,12^ setting in motion several initiatives to restore and conserve mangroves globally thus leading to an overall decline in mangrove cover loss^5,13^.

While deforestation is decelerating globally, the rate of mangrove loss in the SIDS (Small Island Developing States), where 11% of the planet’s mangroves currently grow, seems to increase alarmingly^14^.Vegh et al.^14^ further state that despite SIDS’ efforts to restore and conserve mangrove forests, the lack of documented cases studies, best practices for conservation, weak governance and institutions, unreliable data and the lack of capacity and resources to produce effective forest monitoring data, result in mangroves being sidelined in national agendas. As Bunting et al.^15^ remark, right now, we need to comprehend the changing extent of mangroves because of the role they play in carbon capture, ecosystem services and biodiversity preservation, and even more so for the SIDS. In this respect, remote sensing approaches have been widely applied in studies of mangroves from species mapping^16^, estimating aboveground mangrove biomass^17^ to finding best spots for mangrove planting^18^. As mangroves grow in locations that are often inaccessible, remote sensing provides numerous benefits over traditional field surveys such as analysis of large expanses of forests, cost effectiveness and rapidity of surveys. Mapping the natural extent of mangroves allows for quantifying the services they provide such as carbon sequestration and coastal protection for assets; it also enables the assessment of threats like deforestation, land use change and climate change while long term monitoring studies provide data on the evolutionary dynamics of mangroves over time^19^.

As a signatory to the Paris Agreement, Mauritius identified mangrove restoration as a key measure for climate change mitigation with the added benefits of adaptation from climate change induced externalities in its Nationally Determined Contributions. So, it is of utmost importance to investigate into new geo-spatial technologies and techniques to monitor mangrove areas as concrete actions to maintain and protect coastal habitats are key in climate change adaptation as well as to ensure the livelihoods and well-being of coastal communities^20,21^. Currently, there is a dearth of studies on the application of remote sensing techniques to map and monitor mangroves on islands. The aim of this study was thus to assess remote sensing techniques and GIS (Geographic Information Systems) as tools for monitoring mangroves in island environments. While existing literature extensively covers mangrove studies utilizing remotely sensed data, image processing typically relies on scripting to automate tasks. This study however employs ArcGIS software, which is either free, or licensed to research bodies like universities, has a user-friendly interface and does not necessitate coding expertise for image processing. But due to the lack of historical data on mangrove cover in Mauritius, satellite imagery over the decadal scale (2013 to 2023) was deemed appropriate to carry out the assessment using Random Trees (RT), a machine learning classification algorithm, embedded in ArcGIS Pro. The present research work thus adds to the ongoing research on mangroves at both the regional and international level to help support action plans to conserve mangroves to increase climate change resilience.

## Materials and methods

### Data collection

**Figure 1:**
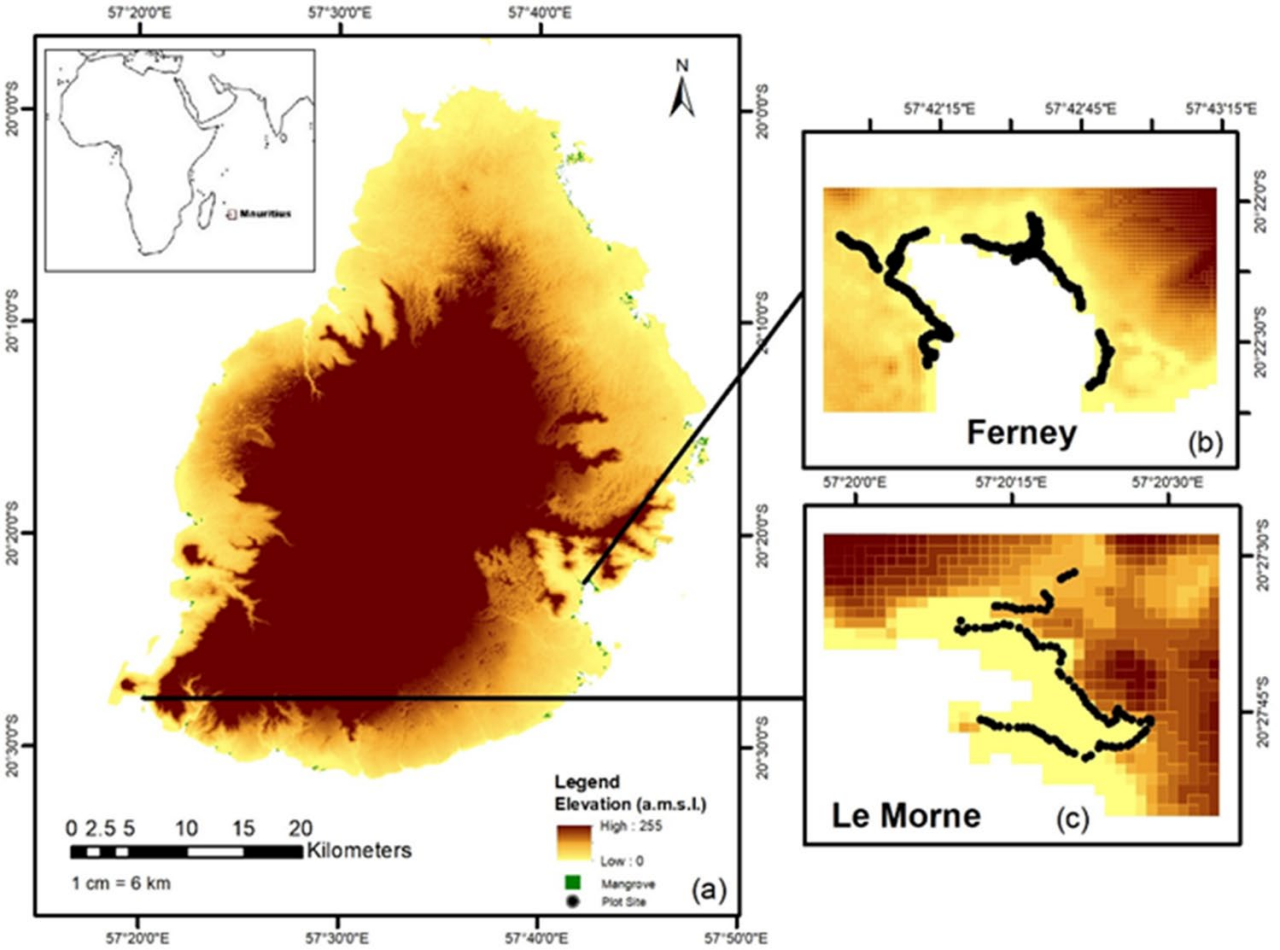
(**a**) Case study area Mauritius in relation to Africa; hillshade and elevation map of the island with study sites Ferney and Le Morne from JAXA (Japan Aerospace Exploration Agency) and mangrove distribution around the island. (**b**) Site location of Ferney and GPS points of plots survey. (**c**) Site location of Le Morne and GPS points of plots survey.

Field surveys were conducted in Mauritius, a SIDS located some 900 km off the east of Madagascar in the southwest Indian Ocean centred on GPS points 20°15′ S and 57°35′ E, to acquire GPS coordinates on mangroves for mapping, training and testing the model. The sites selected were Le Morne (20°27′ S and 57°20′ E) a relatively new planted area found in the south of Mauritius, and Ferney (20°22′ S and 57°42′ E) an aged mature area located on the east coast. The sites were surveyed on the 12th and 13th of August 2023 and since the mangroves are dense and difficult to penetrate, mapping was conducted on the landward side of the forests (refer to Fig. 1). To determine which transect locations were the most suitable, images from Google Earth Pro were examined. GPS coordinates were collected using the mobile application GPS Waypoints version 3.10 with an accuracy of 5 m that has been created by Bluecover technologies, a specialist in geo-location services. Paths were created along the mangrove forests using the mobile application, saved as kml extension and uploaded to ArcMap Desktop (ESRI, Redlands, California). 101 GPS points were collected at Le Morne mainly along the coast (refer to Fig. 1) as tidal level was high that day and inner mangroves could not be penetrated. At Ferney, old mangroves grow along the banks of Riviere Champagne’s estuary. The trees are densely interconnected and the soft muddy substrate made the forest difficult to access, especially along the banks but on the seaward side, younger mangroves grow in the soft muddy substrate. 163 GPS points were collected mainly at the back of the forest, near the mouth of the river and along tidal flats (refer to Fig. 1).

To monitor spatiotemporal change, the decadal scale (2013–2023) was deemed suitable as per this study. Field^22^ in fact states that while a three to five year monitoring scale is often recommended in small-scale restoration projects, a ten-year scale is often more realistic. However, as historical data on mangrove cover was not available for both sites, images from Google Earth Pro were used to map mangroves for the year 2013. Since mangroves have a distinct spectral signature^23^, they were picked accurately at an observer height of 400 m m.a.s.l. A note of caution is added here that mapping from Google Earth Pro based on observer accuracy could have introduced errors into the model. It is thus suggested that further studies consider historical data from topographic, land use and vegetation maps, herbarium records or past studies (if available) for more accurate and reliable results.

### Remote sensing analysis

In general, tropical regions such as Mauritius experience frequent cloud cover which poses a challenge for remote sensing analysis as it obscures satellite imagery. Nevertheless, freely available medium resolution SPOT-5 and Sentinel 2A images were retrieved from their respective repositories. For 2013, SPOT-5 (Satellites Pour l'Observation de la Terre) multispectral images dated 01 March 2013 with minimum cloud cover and a spatial resolution of 10 m were retrieved from the CNES’s Spot World Heritage Programme. For 2023, Sentinel 2A MSI (Multi Spectral Instrument) Level 2A images taken on 02 June 2023 were retrieved from the European Space Agency Copernicus Open Access Hub with minimum cloud cover. While Spot’s mission ended in 2015, Sentinel did not have suitable images for 2013, necessitating the use of two different types of imagery for the assessment. The varying spectral resolutions of these images could have thus impacted on the classification results. Future studies should consider using one type of satellite imagery for more accurate results.

The four bands (B1, B2, B3 and B4) of the SPOT 5 image with a resolution of 10 m were used for the analysis. For the Sentinel image, ten bands were selected (B2, B3, B4, B5, B6, B7, B8, B8a, B11 and B12). To increase the spatial resolution of the bands to 10 m, the 20 m bands were resampled to 10 m using the nearest neighbour resampling technique in ArcGIS (version 2.8.4, ESRI California). The final products of both Spot and Sentinel images were then projected to UTM 40S which preserves the area measure and where the pixels are also addressed as map coordinates rather than pixel and line numbers^24^.

Next, four vegetation indices commonly used in mangrove studies were calculated to extract spectral features from the images as listed below including their formulae:

- NDVI (Normalized Difference Vegetation Index) = (B8 – B4)/(B8 + B4)
- EVI (Enhanced Vegetation Index) = 2.5 × (B8 – B4)/(B8 + 6B4 – 7.5B2 + 1)
- GNDVI (Green Normalized Difference Vegetation Index) = (B8 – B3)/(B8 + B3)
- ReNDVI (Red Edge Normalized Difference Vegetation Index) = (B6 – B5)/(B6 + B5)

For SPOT-5 image analysis, vegetation spectral features were extracted with bands having the same wavelength as the Sentinel bands as is common. PCA was then conducted on ArcMap to remove redundant spectral data from the multiband datasets to retain smaller datasets containing only important spectral data. Four textural variables namely correlation, entropy, homogeneity and mean were extracted using the Gray Level Co-Occurrence Matrices (GLCM) on SNAP (SeNtinel Application Platform) Destktop implementation version 9.0.0. In this study, the GLCM statistical technique was applied using a window size of 9X9 cropped to the study sites, a probabilistic quantizer and quantization levels of 32 and ALL angle to derive the correlation which measures the joint probability occurrence of specified pixel pairs, entropy which measures the degree of disorder of the GLCM, homogeneity which computes how homogenous the image is and mean which weighs the pixel value using the frequency of occurrence of neighbouring pixels. The resulting outputs were stacked in ArcMap to form a 12 feature composite SPOT-5 image (4 spectral bands, four vegetation indices, four GLCM features each in PC1) and a 26 feature composite image for Sentinel 2A (10 spectral bands, four vegetation indices, four GLCM features each in PC1, PC2 and PC3). Supervised object based classification was then conducted on ArcGIS Pro using the Random Trees classifier. Random Trees uses multiple decision trees that are trained with small numbers of the same training data and where the majority vote of the trained trees decide on the output class. Random Trees was chosen for the analysis as it is claimed to use the same algorithm as Random Forest^25^, one of the most commonly used classifier in mangrove mapping and monitoring. Since the aim of the study was to evaluate the potential of remote sensing tools for management purposes, only one classifier was deemed necessary. Future studies should investigate on the performance of different classifiers for best mapping mangroves in Mauritius. A maximum of 50 trees with a maximum tree depth of 30 and a maximum number of 1000 samples per class was applied. Chromaticity, colour, mean digital number and standard deviation were selected as segment attributes. Finally, stratified Random Strategy, a commonly applied statistical technique that creates points which are randomly distributed within each class and where each class contains a number of points proportional to its relative area, was applied to assess the accuracy of the classification. Error mask images where pixels were incorrectly classified in each class were calculated in a confusion matrix. Based on these confusion matrices, kappa analysis was conducted to assess the spectral signatures of the captured data. Training and testing data used for the classification were resampled to a 70% training and 30% testing split as recommended by^26^ and datasets for each site were resampled 10 times to obtain the best results.

## Results

### Field data observations

**Figure 2:**
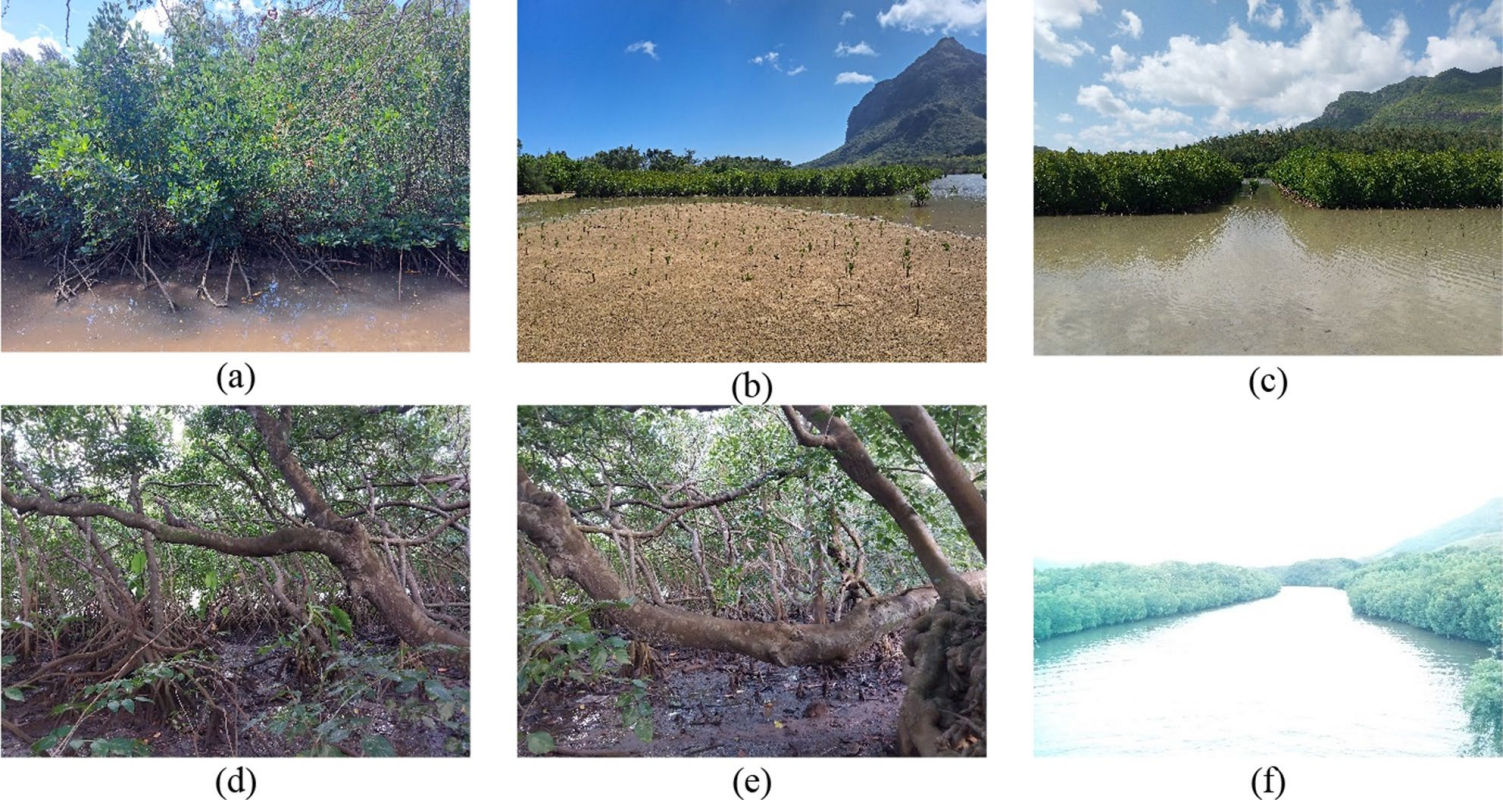
(**a–c**) Mangrove forest at Le Morne. (**a**) Young adults of *Rhizophora* species growing in the sandy substrate (**b**) Seedlings planted in sandy substrate with young adults at the back. (**c**) Young adults growing in quadrats. (**d–f**) Mangrove forest at Ferney. (**d**) Mature *Rhizophora* species growing in the muddy substrate. (**e**) *Brugeira* species with exposed pnematophores at low tide. (**f**) Mangroves growing on the banks of Riviere Champagne’s estuaries. Photographs were taken by first author during the data collection in 2023.

Both mangrove species *Rhizophora mucronata* and *Bruguiera gymnorrhiza* were encountered at the two selected sites, though *Rhizophora* was the dominant species. At Le Morne, the landward fringe consisted mainly of old, matured mangrove stands that were impenetrable as a result of the interconnection of prop roots and branches. Along the sandy seaward size, where mangrove plantation efforts were underway, adult trees and seedlings were observed growing in quadrats as depicted in Fig. 2. *Bruguiera* was present at Ferney which featured aged trees with seedlings and young adults scattered across open mudflats.

### Feature analysis

When it comes to satellite image analysis, texture is a key component in identifying objects on an image. PCA is a popular technique that reduces the dimensionality of a dataset while preserving as much variability as possible. Table 1 shows the percent and accumulative of eigenvalues for Spot and Sentinel images for both sites.

**Table 1:** Eigenvalues for PC1 of SPOT-5 images and PC1, PC2, and PC3 of Sentinel 2A images for Le Morne and Ferney.

Site	PC Layer	Percent of Eigenvalues (%)	Accumulative of Eigenvalues (%)
Le Morne	SPOT-5 2013		
	1	100	100
	Sentinel 2A 2023		
	1	93.38	93.38
	2	5.26	99.06
	3	1.36	100
Ferney	SPOT-5 2013		
	1	100	100
	Sentinel 2A 2023		
	1	90.44	90.44
	2	7.41	96.51
	3	2.16	100

For Spot images, only PC 1 was extracted which represents 100% of the images. For Sentinel images, PC1, PC2 and PC3 were extracted totalling 100% coverage of the images. This feature extraction was critical in increasing the visibility of the satellite images making certain details more noticeable. The eigenvalues for both sites describe the percentage of variance within each principal component where higher eigenvalues as sums of eigenvalues represent more data in that principal component. Since only PC1 was extracted for Spot images, both the percent and accumulative of eigenvalues totalled 100%. But for Sentinel images, PC1 captured the largest amount of data for both sites, followed by PC2 and PC3 as has been noted in other studies such as Estornell et al.^27^, Mohammadpour et al.^28^ and Chen^29^. According to Wiegleb^30^, PCA is ecologically appropriate in spatio-temporal analysis studies such as the work by Lasaponara^31^.

### Image classification and change analysis

**Figure 3:**
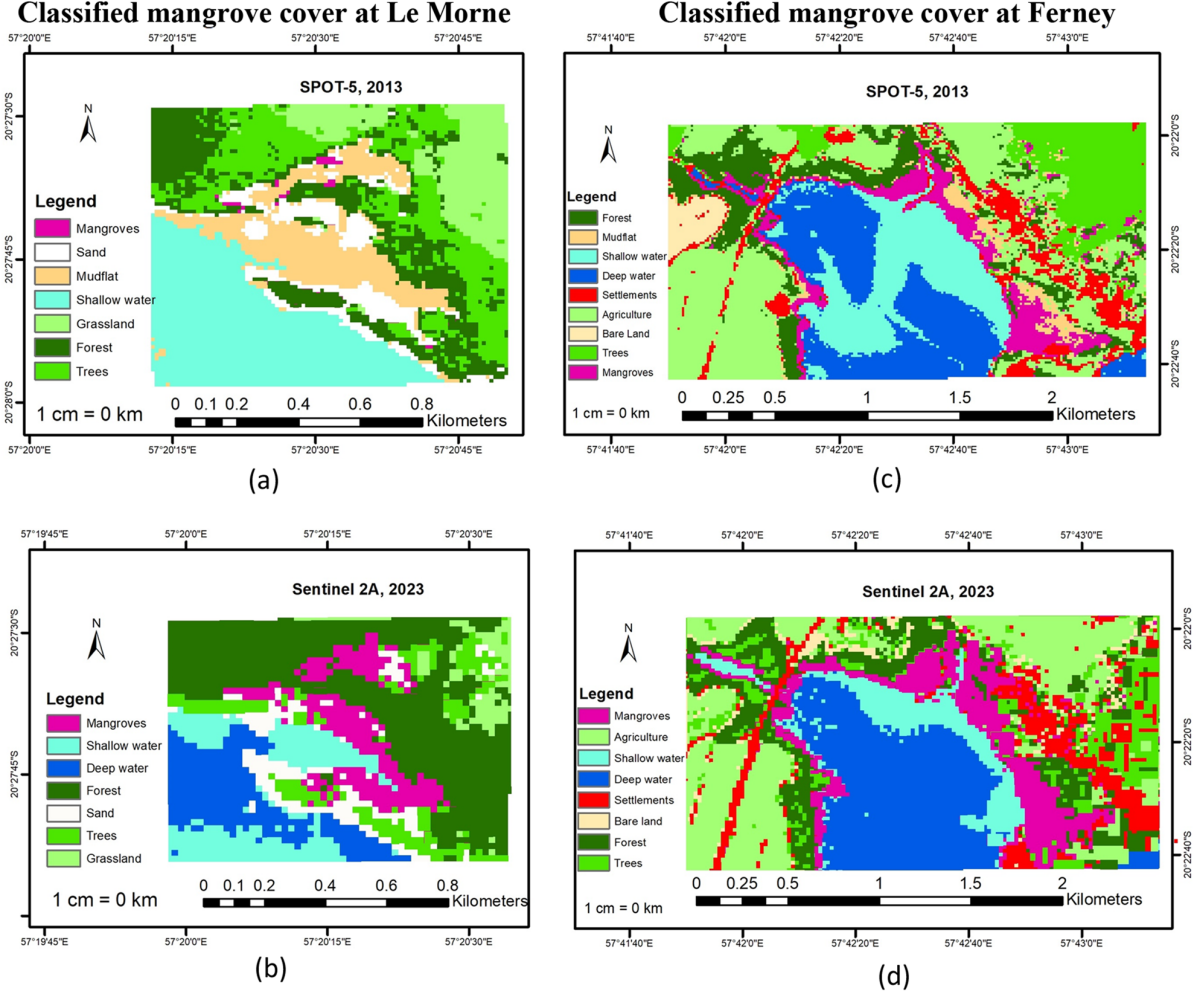
Classified mangrove cover at selected sites (**a**) Mangrove distribution at Le Morne in 2013 based on SPOT-5 image classification. (**b**) Mangrove distribution at Le Morne in 2023 based on Sentinel 2A image classification. (**c**) Mangrove distribution at Ferney in 2013 based on SPOT-5 image classification (**d**) Mangrove distribution at Le Morne in 2023 based on Sentinel 2A image classification.

Figure 3 illustrates the classified mangrove cover at Le Morne and Ferney for the years 2013 and 2023. Land cover classes for Le Morne are (a) mangroves with a distinct spectral signature, (b) deep water representing deep sea, (c) shallow water representing shallow water areas, (d) forest representing dense, closely conglomerated trees, (e) trees referring to isolated clusters of trees, (f) sand representing open sandy areas and (g) grassland for open grassland. For Ferney, the classes are (a) mangroves with a distinct spectral signature, (b) deep sea representing deep water areas, (c) shallow water representing shallow water areas, (d) forest representing dense, closely conglomerated trees, (e) trees referring to isolated trees, (f) mudflat referring to visible open muddy areas g) settlements referring to houses, buildings and roads and (h) agriculture representing planted land and (i) bare land representing open bare land.

**Figure 4:**
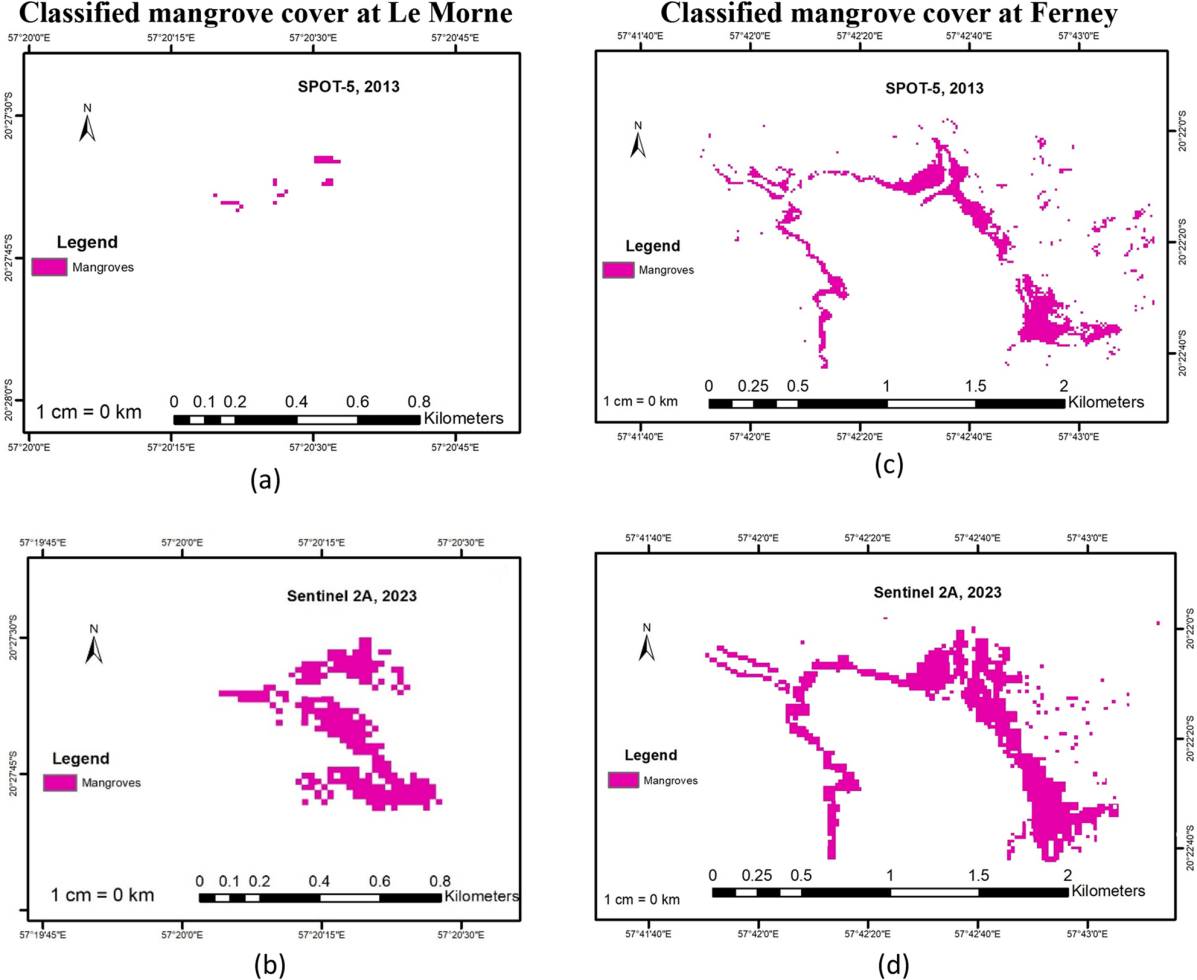
Classified mangrove cover with feature masks applied (**a**) Mangrove distribution at Le Morne in 2013 based on SPOT-5 image classification. (**b**) Mangrove distribution at Le Morne in 2023 based on Sentinel 2A image classification. (**c**) Mangrove distribution at Ferney in 2013 based on SPOT-5 image classification. (**d**) Mangrove distribution at Le Morne in 2023 based on Sentinel 2A image classification.

Figure 4 represents the classified mangrove cover at Le Morne and Ferney for 2013 and 2023 with feature masks applied to exclude all other classes. It can be seen that at Le Morne, the mangrove cover was quite small in 2013 but the area increased significantly in size for 2023 based on RT classification. For Ferney, mangroves covered almost all of the site’s coast in 2013 while a subtle increase in coverage can be noted for 2023 based on RT classification.

**Figure 5:**
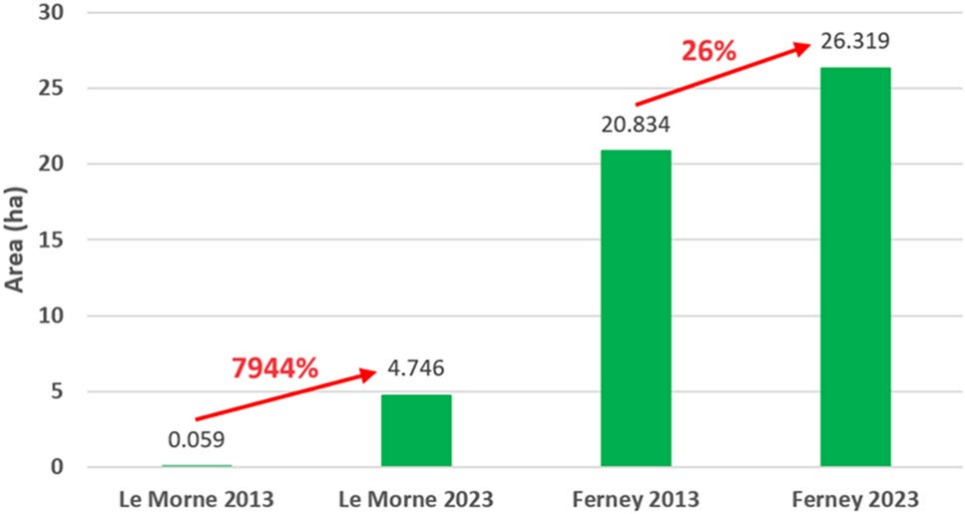
Mangrove cover change at Le Morne and Ferney during the period 2013 and 2023, mangroves increased from 0.059 ha in 2013 at Le Morne to 4.75 ha representing an increase of 7944% and from 20.834 ha to 26.319 ha at Ferney representing an increase of 26%

Figure 5 shows the area change of mangrove forest over the decade for Le Morne and Ferney. Mangrove area increased from 0.059 ha in 2013 at Le Morne to 4.75 ha in 2023 representing an increase of 7944%. At Ferney, mangroves also increased from 20.834 ha in 2013 to 26.319 ha in 2023 representing an increase of 26%. As illustrated by Figs. 3 and 4, a significant increase can be observed at Le Morne due to active plantation programmes. Nevertheless, a 26% increase is observed at Ferney as a result of natural regeneration.

### Accuracy assessment

The confusion matrices depict the accuracy levels of the image classification exercise using the RT classifier in ArcGIS Pro based on the kappa coefficient of agreement (refer to Tables 2, 3, 4 and 5). All images had kappa values of over 0.9 which are considered to be excellent agreement between observed and expected accuracy^32^. At Le Morne, the kappa values were 0.91 and 0.93 for the classified 2013 and 2023 images respectively (refer to Tables 2 and 3); for Ferney the kappa values were 0.94 and 0.91 for the classified 2013 and 2023 images respectively (refer to Tables 4 and 5). The general confusion in modeling was seen to be between mangroves, forest and trees for all images.

**Table 2:** Confusion matrix with classes mangroves, sand, mudflat, shallow water, grassland, forest and trees for the SPOT-5 image 2013 at Le Morne.

Confusion matrix										
SPOT-5 2013, Le Morne										
Class	Mangroves	Sand	Mudflat	Shallow water	Grassland	Forest	Trees	Total	U.accuracy	Kappa
Mangroves	80	0	0	0	1	2	1	84	0.95	0
Sand	0	9	1	1	0	0	0	11	0.81	0
Mudflat	0	1	23	0	0	0	0	24	0.96	0
Shallow water	0	0	1	15	0	0	0	16	0.94	0
Grassland	2	0	0	0	31	1	0	34	0.91	0
Forest	3	0	0	0	1	43	1	48	0.9	0
Trees	1	0	0	0	1	1	35	38	0.92	0
Total	86	10	25	16	34	47	37	255	0	0
P.Accuracy	0.93	0.9	0.92	0.94	0.91	0.9	0.95	0	92.55	0
Kappa	0	0	0	0	0	0	0	0	0	0.91

**Table 3:** Confusion matrix with classes mangroves, sand, mudflat, deep water, shallow water, grassland, forest and trees for the Sentinel 2A image 2023, at Le Morne.

Confusion matrix										
Sentinel 2A 2023, Le Morne										
Class	Mangroves	Sand	Deep water	Shallow water	Grassland	Forest	Trees	Total	U.accuracy	Kappa
Mangroves	98	0	0	0	0	5	4	107	0.92	0
Sand	0	25	1	0	0	0	0	26	0.96	0
Deep water	0	0	17	1	0	0	0	18	0.94	0
Shallow water	0	0	0	12	0	0	0	12	1	0
Grassland	0	0	0	0	23	0	0	23	1	0
Forest	2	0	0	0	1	60	1	64	0.94	0
Trees	1	0	0	0	1	1	60	63	0.95	0
Total	101	25	18	13	25	66	65	313	0	0
P.Accuracy	0.97	1	0.94	0.92	0.92	0.91	0.92	0	94.2	
Kappa	0	0	0	0	0	0	0	0	0	0.93

**Table 4:** Confusion matrix with classes mangroves, bare land, mudflat, shallow water, deep water, settlements, agriculture, forest and trees for the SPOT-5 image 2013, at Ferney.

Confusion matrix												
SPOT-5 2013, Ferney												
Class	Mangroves	Bare Land	Mudflat	Shallow water	Deep water	Settlements	Agriculture	Forest	Trees	Total	U.accuracy	Kappa
Mangroves	110	0	0	0	0	0	0	3	3	116	0.95	0
Bare land	0	3	0	0	0	0	0	0	0	3	1	0
Mudflat	0	0	13	0	0	0	0	0	0	13	1	0
Shallow water	0	0	0	14	0	0	0	0	0	14	1	0
Deep water	0	0	0	0	12	0	0	0	0	12	1	0
Settlements	0	0	0	0	0	50	0	0	0	50	1	0
Agriculture	0	0	0	0	0	0	25	0	0	25	1	0
Forest	1	0	0	0	0	1	0	20	1	23	0.87	0
Trees	1	0	0	0	0	1	0	2	24	28	0.86	0
Total	112	3	13	14	12	52	25	25	28	284	0	0
P.Accuracy	0.98	1	1	1	1	0.96	1	0.8	0.86	0	95.4	0
Kappa	0	0	0	0	0	0	0	0	0	0	0	0.94

**Table 5:** Confusion matrix with classes mangroves, agriculture, shallow water, settlements, bare land, deep water, forest, and trees for the Sentinel 2A image 2023, at Ferney.

Confusion matrix											
Sentinel 2A 2023, Ferney											
Class	Mangroves	Agriculture	Shallow water	Settlements	Bare land	Deep water	Forest	Trees	Total	U.accuracy	Kappa
Mangroves	115	0	0	0	0	0	5	2	122	0.94	0
Agriculture	0	18	0	0	0	0	0	0	18	1	0
Shallow water	0	0	15	0	0	0	0	0	15	1	0
Settlements	0	0	0	60	0	0	0	0	60	1	0
Bare land	0	0	0	0	3	0	0	0	3	1	0
Deep water	0	0	0	0	0	12	0	0	12	1	0
Forest	2	0	0	4	0	0	23	2	31	0.74	0
Trees	1	0	0	2	0	0	1	27	31	0.87	0
Total	118	18	15	66	3	12	29	31	292	0	0
P.Accuracy	0.97	1	1	0.91	1	1	0.79	0.87	0	93.5	0
Kappa	0	0	0	0	0	0	0	0	0	0	0.91

## Discussion

Johnson et al.^33^ remark that the application of remote sensing and GIS technologies on island states depend upon the availability of accurate spatial information together with non-spatial data to provide relevant results to end users. At this point, sparse studies are available on the use of remote sensing techniques to monitor ecological systems in Mauritius including Koener’s et al.^34^ analyses on various classification approaches applied to Sentinel 2A imagery to map an alien invasive plant species in the tropical forests of Mauritius, Doodee’s et al.^35^ assessment of mangrove growth rate using Google Earth Pro satellite imagery and Sunkur and Mauremootoo^36^’s present, past and future distribution of the invasive alien species *Vachellia Nilotica* on Rodrigues Island. In general, for island nations including Mauritius, remote sensing analysis of ecosystems is still in its infancy due to several challenges such as the lack of human expertise as listed in Table 6; but if these challenges are addressed appropriately, remote sensing tools can be key in mapping island wise mangroves, monitoring changes and extracting biophysical parameters for in-depth studies.

**Table 6:** The different aspects of remote sensing technology including their advantages, disadvantages and mitigating measures to monitor mangroves in the context of Mauritius.

Aspect	Advantage	Disadvantage	Mitigation	References
Wide coverage	Cover wide and remote areas which can help to understand distribution of mangroves across large scales, identify vulnerable sites and apply site specific conservation strategies	Can be difficult to capture complex terrains, for example Rodrigues Island has an extensive cover of mangroves but it is not covered by the Global Mangrove Watch database	With the launch of the Mauritian 1U CubeSat (MRIC Sat-1) in 2021, data on local ocean resources is being captured which can be used in local mangrove monitoring studies	^37^
Resolution	For preliminary assessments, low and medium resolution imagery are suitable to set baselines	Certain remote sensing technologies cannot achieve high spectral resolution and are often weather dependent	Depending on the aim of the project, low and medium resolution imagery support spatio-temporal mapping and high resolution imagery provide more in depth data such as biomass, carbon stock etc	^29,38^
Cost	Low and medium resolution images are free as well as many satellite imagery processing platforms such as GEE, QGIS, SNAP which can be used in spatio-temporal studies and compare classification algorithms for best results	High resolution images for Mauritius are very expensive in the range of $312.50 to $812.50 for 25 square kilometres of coverage; in the case of Mauritius, due to the very small spatial coverage of mangroves, high resolution imagery would be ideal to extract additional data such as ecosystem dynamics, species interaction etc	At the government level, under the Ministry of Finance, the National Environment and Climate Change Fund (NECCF) prioritizes climate change projects in Mauritius which would provide funding for remote sensing analysis project on mangroves to increase climate change resilience; NGOs working on mangroves can also leverage funding from other regional and international initiatives such as the Mitsui O.S.K. Lines, Ltd (MOL)	^39,40^
Revisit frequency	Regular and consistent revisit times allow consistent monitoring over time e.g. Sentinel twin satellites have a 5-day revisit time; long term datasets can thus help in tracking changes over different time scales, assess the impacts of external disturbances such as cyclones, evaluate the effectiveness of management efforts and make informed decisions	Compiling time scale datasets takes time and is not always available for all areas such as a specific coast of Mauritius; can overlook certain critical events such as disturbance or even illegal logging	Data from different sensors (with different spectral and spatial resolutions) can be used while applying image specific processing techniques to maintain accuracy	^41,42^
Type of imagery (multi spectral and hyperspectral imaging—MSI, HSI)	MSI and HSI both have their own applications depending on the aim of the project but in general while MSI has wide applications and are cost effective HSI provides detailed spectral data and allows for quite accurate characterization of objects in mangroves studies	MSI has limited spectral detail and cannot capture fine objects while HSI is complex to manipulate, is less readily available and expensive	The choice between the type of imagery is dependent on the goal of the project, the level of detail required and budget constraints for e.g. MSI can be used to assess mangrove health and HSI to model the impacts of pest and disease on leaf traits; the European Space Agency Third Party Missions Collections provides a range of MSI/HSI images for free upon request	^43–45^
Data processing	Most satellite images are pre-processed for analysis; data processing and analysis using certain software such as ArcGIS is relatively easy and straightforward as the user interface is user friendly with help snippets at each stage of processing	Certain images require complex pre-processing before use; for most platforms such as GEE, programming skills are required to automate tasks like image acquisition and processing	Cloud computing and big data technologies can facilitate collaboration with other researchers by providing shared workplaces and tools for data analysis irrespective of geographical locations	^46,47^
Ground truthing	Collection of on-the-ground data complements mangrove studies as it ensures that real-world conditions are accurately represented especially for localised sites which can then be used to identify vulnerabilities to climate change more accurately thus supporting site specific adaptive strategies	Can be time intensive and expensive especially in remote areas where there can further be logistical challenges and safety considerations for field researchers; valid historical points are often not available	Engage stakeholders, especially local communities, in participatory approaches to gather ground truth data; drones can also be used to capture aerial images for validation, for instance the Mauritius Oceanographic Institute (MOI) and National Parks and Conservation Service (NPCS) have drones which can be used for such purposes if requested	^48,49^

Monitoring mangroves using remote sensing techniques can substantially increase a nation’s resilience to climate change. According to the Blue Carbon Initiative, coastal blue carbon ecosystems such as mangroves provide a wide array of mitigation, adaptation and resilience benefits to tackle climate change including coastline protection from storm surge and sea level rise, sediment trapping, erosion prevention, nutrient recycling, coastal water quality regulation, habitat provision for endangered and commercially important species, sequestering and storing large amounts of carbon, ensuring livelihood continuity and food security^50^. Remote sensing techniques can help support a number of mitigation and adaptation strategies including:

1. Early detection of changes: changes in mangrove ecosystems such as illegal logging, coastal development and degradation can be easily detected allowing authorities to take timely action^51–53^.
2. Vulnerability assessment: mangroves’ vulnerability to climate related impacts such as sea level rise and storm surge can be assessed and this information can be used to guide adaptive management strategies^54^.
3. Mapping coastal dynamics: remote sensing tools provide critical information on coastal changes such as erosion and changes in shoreline which can be used to plan and implement strategies to protect mangroves and coastal areas against climate change induced impacts^42^.
4. Carbon sequestration: satellite imagery can help quantify mangrove biomass and the amount of carbon stored^55,56^.
5. Community engagement: satellite imagery can be important visual tools to engage local communities in participating in the monitoring and management of mangroves^57^.

Likewise, the results of this study underscore the efficacy and dependability of remote sensing techniques in monitoring mangroves, thereby enhancing Mauritius’ capacity to withstand the impacts of climate change. SPOT-5 imagery has been used widely in mangrove monitoring studies until the satellites were decommissioned in 2015 such as the works by Valderrama-Landeros et al.^58^ as reported by Valderrama-Landeros et al.^59^ and Pham et al.^60^ and today SPOT-5 archives remain an important tool for historical mapping of ecosystems as presented in this study. Sentinel images are more widely used in mangrove mapping and monitoring studies^61–63^ as they are more easily accessible and up to date and often perform better in classification compared to other medium resolution images such as Landsat 8 OLI due to their higher spatial resolution^64^. Using SPOT-5 2013 and Sentinel 2A 2023 imagery, it was observed that mangroves cover at both Le Morne and Ferney were distinctly identified on the images due to the particular spectral signature of mangroves largely based on their MSI data. Tran et al.^65^ further remark that *Rhizophora* reflectance is seen to be higher in the NIR region for Sentinel 2A imagery with a rapid rise at the red-edge. This was a critical element to distinguish mangroves on the landward side at both Le Morne and Ferney which are mixed with terrestrial trees. By applying the classic vegetation indices NDVI, EVI, GNDVI and ReNDVI which are all based on the red-edge bands of the Spot and Sentinel images, detection of the spectral signature of mangroves was enhanced. Likewise feature analysis using GLCM features and applied to PCA transformation refined the model by discriminating between different land cover types such as mangroves, grassland and settlements^29,65,66^. Thus for Le Morne, a site where active planting has been ongoing since 2008, the spatial distribution of mangroves was easily mapped as the planted area was homogenous except on the landward side. In contrast, at Ferney, mangroves are matured and interspersed with terrestrial trees along the landward side of the river banks. In this case, vegetation indices played a crucial role in distinguishing mangroves from the terrestrial canopy, resulting in accurate forest mapping.

As it is, mangrove mapping and monitoring can be carried out using a wide variety of techniques such as Google Earth Engine (GEE) as reported by^67^. Such cloud computing platforms automate several tasks such as retrieving satellite imagery directly which are stored in the cloud and facilitating data access and sharing^23^. However, these platforms are code based requiring computer language programming skills for remote sensing analysis. In the context of Mauritius, which can be expanded to other island states, environmental scientists and researchers are typically trained in theoretical environmental sciences and basic computer science knowledge with limited exposure to computer programming. Users who are not familiar with coding may face a steep learning curve to use these platforms. To overcome this particular matter, the present study used ArcGIS software to conduct the remote sensing analysis. While ArcGIS may not always receive much attention in remote sensing analysis as other specialised software, it remains a powerful tool to conduct remote sensing studies. It has a user friendly interface allowing researchers and practitioners to easily access and analyse remote sensing data without programming knowledge, offers a comprehensive set of tools including image pre-processing, classification and spatial analysis and integrates remote sensing data with other geospatial information.

To be sure, remote sensing analysis and digital image classification can be quite challenging and classification accuracy typically depends on spatial, spectral and textural features of an image as well as the type of leaning used^66^. Supervised, object based classification where the user identifies objects based on the same set of pixels was key in this study in identifying the different classes. Each image was different from one another with different classes and so it was crucial to identify each class separately. For example, in the Spot images of both Le Morne and Ferney, mudflats were visible but not in the Sentinel images. Outputs were then validated through ground truth data, a key step in evaluating the classification accuracy. Nonetheless confusion still arose during the classification process especially between mangroves, trees, forests and even grassland in both the Spot and Sentinel images. It is believed that errors were introduced during modeling as a result of class inseparability with an overlap in mangrove, forest, trees and grassland classes due to their similar spectral resolutions. Else, inappropriate data pre-processing for both Spot and Sentinel images or even the use of Random Trees classifier for the analysis could have affected the analysis. It is worth to add here that the pixelated appearance of the classified maps is due to the small extent of the study areas (refer to Figs. 2 and 3).

As for the spatiotemporal change in mangrove cover at the selected sites, a notable increase in mangrove cover can be observed at both sites. Since the study focused on mangrove change, all other class change (shallow water, deep water, grassland, agriculture, mudflats, bare land, sand, settlements, trees and forests) were excluded. The present study illustrates clearly how using remote sensing techniques, mangrove cover change can be tracked and plantation efforts monitored. Following mass deforestation of mangroves in Mauritius during the period 1987–1994 (from 2000 to 1400 ha) for boat passage, construction and for firewood, a mangrove propagation programme started in 1995 and is still ongoing^68^. Since 2009, the non-governmental organization Association pour le Développement Durable (ADD) supported by the European Union, the Ministry of Finance and the Mauritius Commercial Bank Forward Foundation have planted 50,000 mangrove seedlings at Le Morne^69^ which today represent one of the largest mangrove areas in Mauritius. As it is, ADD claims that the mangrove cover at Le Morne is today approximately 5 ha^70^ which tally with the results obtained in this study. Likewise, the major class change observed here is from mudflat to mangroves which is where mangroves have been planted during the study period (refer to Fig. 2). At Ferney, it can also be observed that mangroves expanded from 20.834 ha to 26.319 ha representing an increase of 26%. Ferney is a mature site where mangroves basically grow on the banks and mouth of Riviere Champagne. What is evident from the classification is that during this ten-year period, mangroves expanded over mudflats on the landward side of the river mouth with minimal change within the river banks and an observable thin stretch towards the seaward region. The change observed here is a natural establishment where seeds fall into water, move with tides, get deposited in muddy substrate, form roots, grow and form dense thickets over the years. Baba et al.^71^ report that mangroves at Ferney have developed and established over several hundred years based on C-14 dating collected from the site, with mature old trees at the back and seedlings towards the seaward area. This would explain why the classification resulted in the seaward expansion with respect to seedling growth as well as class confusion with forest as the mangrove trees become denser and more tightly packed together. It can be assumed that the results of this study also match Baba et al.’s^71^ observations. Therefore, it can be concluded that remote sensing and GIS, including the RT classifier, are powerful tools to map and monitor mangroves spatially and temporally even with freely available medium resolution satellite imagery and for very small areas. More specifically, the technique applied in this study based on the use of medium resolution satellite imagery of SPOT-5 and Sentinel 2A, coupled to vegetation indices plus GLCM and PCA data resulted in an accurate mapping of mangroves for the years 2013 and 2023 similar to other studies such as Chen’s^27^. The present study demonstrates how remotely sensed data when combined with GIS mapping tools can be an efficient technique to monitor mangrove change across landscapes thereby providing invaluable insights, that could otherwise be unattainable, thus helping in informed decision making to increase climate change resilience. More importantly, the study demonstrates the potential for practical, precise, repeatable and cost-effective assessment of mangrove distribution over time, offering reliable monitoring data to support management efforts. As highlighted by Leal and Spalding^5^, in order to meet the targets of the UN Decade on Restoration, the Bonn Challenge and even the United Nations’ SDGs (Sustainable Development Goals), there is an urgency to improve documentation, monitoring, evaluation and outcomes of mangroves studies globally such as provided in this case study.

### Limitations

The main limitation in this study was the lack of historical data on mangrove coverage in Mauritius. Ground truthing is key in validating remotely sensed products and as a result, the earliest (2013) detectable mangrove cover on Google Earth satellite images were used. Further studies could consider historical data from topographic, land use and vegetation maps, herbarium records or past studies (if available) for more accurate and reliable results. Also, different types of satellite imagery were used for the analysis as SPOT-5’s mission ended in 2015 so Sentinel 2A images were used for current mapping and analysis. Radiometric differences, temporal variations and spectral differences between these two sensors could have affected the classification results. Future studies should consider using one type of satellite imagery for more accurate results such as Sentinel 2018, 2021, 2024 imagery. Finally, Mauritius is located in the tropics which is heavily clouded and is reflected on satellite images; browsing through libraries of images with minimal cloud coverage is time consuming. As a result, the decadal scale was deemed appropriate for the assessment but if cloud free images are readily available, future works could consider varying the time scales for more reliable results and in depth analysis.

## Conclusions

Remote sensing and GIS technology has been used globally to map and monitor mangrove forest from China to Mexico. But for island nations using such technology, especially when it involves machine learning, has been challenging particularly due to limited human expertise. This study thus filled in this gap by providing an innovative method to map and monitor mangroves that is feasible, free and does not require complex coding understanding. Medium resolution satellite images of SPOT-5 and Sentinel 2A for the years 2013 and 2023 were retrieved and processed to produce decadal mangrove distribution maps. The analysis was conducted on ArcGIS Pro and the images were classified using the supervised learning algorithm Random Trees. Mangroves were accurately mapped for the time period with kappa values in the 90s and in agreement with other published materials and ground truthing data. It was observed that human driven plantation at Le Morne significantly increased the mangrove cover by 7944% while at Ferney, natural mangrove establishment also increased mangrove cover by 26%. In both cases it was observed that class change was mainly from mudflat to mangrove and confusion in models arose with other vegetation classes probably due to their similar spectral signature. This study not only provides baseline data on the spatiotemporal distribution of mangroves in Mauritius but also demonstrates how remote sensing technologies can be powerful tools to monitor mangroves for better management. Mangroves have been proven to be robust ecosystem based solutions against climate change induced hazards such as higher swell waves. Remote sensing techniques can thus help support mitigation and adaptation strategies by providing key information on mangrove distribution, carbon stock storing capacity, vulnerability assessments and engaging communities to support mangrove management actions. It is thus critical to investigate into novel geospatial techniques and tools to monitor mangrove cover change, be it expansion or decline, so as to prioritize conservation actions and enhance the island’s resilience to climate change.

### Recommendations

Several recommendations pointed out herein can be utilized to achieve more accurate results. The possibility of implementing these recommendations can be investigated in future studies. While the present study demonstrates that medium resolution satellite imagery can be helpful in mangrove and monitoring, it is best to use fine resolution (2.5 m) imagery for analysis of mangroves in Mauritius as the patches are very small in size. High spatial resolution images of Mauritius are freely available at the ESA (European Space Agency) third party mission collections for free upon request. This is an avenue that can be explored for more accurate results for future works. Support Vector Machine (SVM) is another common supervised learning algorithm used in mangrove classification studies and is available in the ArcGIS Pro suite. It would be interesting to study how different classification models (RT, SVM) affect mangrove distribution and identify which one works best for Mauritius. Future studies should also test the feasibility of the GEE platform for mangrove mapping and monitoring using Sentinel-2-time series data and even a combination of Sentinel 1 and 2 to increase the accuracy of forest maps.

The scope of this study was to demonstrate how medium resolution satellite imagery can be used for mangrove mapping and monitoring. With this baseline data, more in depth studies can be conducted such as zonation patterns, species delineation, impact assessments, carbon storage capacity etc. Land use change can also be more thoroughly assessed. Also, using ArcGIS Online, it is possible to upload datasets from remotely sensed products, shapefiles, field survey data in mangrove monitoring projects and share the results with different communities locally and globally. It is strongly recommended to set up such a database for Mauritius, where researchers and scientists can have access to geospatial data and even collect real time field survey data using the mobile application Survey123. It is also possible to predict future mangrove cover change using the free MOLUSCE (Modules of Land Use Change Evaluation) plugin in QGIS. However, the satellite images must be of similar spatial and spectral resolution for an appropriate classification. Hence, upcoming studies could focus on future spatial distribution of mangroves using MOLUSCE.

## Data Availability

All satellite imagery used in this study are open access remote sensing data. SPOT-5 imagery are freely available at the CNES portal https://regards.cnes.fr/user/swh/modules/60 and Sentinel 2A imagery are freely available at the ESA portal https://sentinels.copernicus.eu/. All software used are detailed in the manuscript. GPS coordinates of mangrove location points collected in this study can be made available upon request.
